# Characterization of Cross-Linked Porous Gelatin Carriers and Their Interaction with Corneal Endothelium: Biopolymer Concentration Effect

**DOI:** 10.1371/journal.pone.0054058

**Published:** 2013-01-30

**Authors:** Jui-Yang Lai, David Hui-Kang Ma, Meng-Heng Lai, Ya-Ting Li, Ren-Jie Chang, Li-Mei Chen

**Affiliations:** 1 Institute of Biochemical and Biomedical Engineering, Chang Gung University, Taoyuan, Taiwan, Republic of China; 2 Biomedical Engineering Research Center, Chang Gung University, Taoyuan, Taiwan, Republic of China; 3 Molecular Medicine Research Center, Chang Gung University, Taoyuan, Taiwan, Republic of China; 4 Limbal Stem Cell Laboratory, Department of Ophthalmology, Chang Gung Memorial Hospital, Taoyuan, Taiwan, Republic of China; 5 Department of Chinese Medicine, Chang Gung University, Taoyuan, Taiwan, Republic of China; University of California Merced, United States of America

## Abstract

Cell sheet-mediated tissue regeneration is a promising approach for corneal reconstruction. However, the fragility of bioengineered corneal endothelial cell (CEC) monolayers allows us to take advantage of cross-linked porous gelatin hydrogels as cell sheet carriers for intraocular delivery. The aim of this study was to further investigate the effects of biopolymer concentrations (5–15 wt%) on the characteristic and safety of hydrogel discs fabricated by a simple stirring process combined with freeze-drying method. Results of scanning electron microscopy, porosity measurements, and ninhydrin assays showed that, with increasing solid content, the pore size, porosity, and cross-linking index of carbodiimide treated samples significantly decreased from 508±30 to 292±42 µm, 59.8±1.1 to 33.2±1.9%, and 56.2±1.6 to 34.3±1.8%, respectively. The variation in biopolymer concentrations and degrees of cross-linking greatly affects the Young’s modulus and swelling ratio of the gelatin carriers. Differential scanning calorimetry measurements and glucose permeation studies indicated that for the samples with a highest solid content, the highest pore wall thickness and the lowest fraction of mobile water may inhibit solute transport. When the biopolymer concentration is in the range of 5–10 wt%, the hydrogels have high freezable water content (0.89–0.93) and concentration of permeated glucose (591.3–615.5 µg/ml). These features are beneficial to the in vitro cultivation of CECs without limiting proliferation and changing expression of ion channel and pump genes such as ATP1A1, VDAC2, and AQP1. In vivo studies by analyzing the rabbit CEC morphology and count also demonstrate that the implanted gelatin discs with the highest solid content may cause unfavorable tissue-material interactions. It is concluded that the characteristics of cross-linked porous gelatin hydrogel carriers and their triggered biological responses are in relation to biopolymer concentration effects.

## Introduction

In the field of regenerative medicine, cell sheet engineering is a novel technology for tissue reconstruction without artificial scaffolds using thermo-responsive culture dishes [1]. Okano’s group first proposed the concept of temperature-modulated cell adhesion/detachment using poly(*N*-isopropylacrylamide) (PNIPAAm)-based nanostructured coatings fabricated by electron beam irradiation [2]. They found that the cultivated cells proliferate well on the hydrophobic PNIPAAm matrices at 37°C, and spontaneously detach from the hydrophilic surfaces when the surrounding temperature is reduced to a level below the lower critical solution temperature of thermo-responsive polymer [3]. Due to the avoidance of enzymatic treatment, the tissue-engineered cell sheets harvested from the PNIPAAm coatings are able to retain their cellular activity, organization, function, and extracellular matrix integrity. All of these features are important to maximize graft quality for the patient. Up to now, the transplantable cell sheets have already been investigated for the treatment of diseases from corneal dysfunction to esophageal cancer, tracheal resection, and cardiac failure [4].

For the eyes of recipients with severe bilateral limbal stem cell deficiency, Nishida et al. have presented a strategy that allows carrier-free and sutureless cell sheet transplantation [5]. Although autologous oral mucosa epithelium fabricated from thermo-responsive culture dishes is very effective for ocular surface reconstruction, the occurrence of corneal opacities is closely related to endothelial dysfunction in most patients requiring penetrating keratoplasty [6]. Therefore, we, as others, have focused on the bioengineering of corneal endothelium as tissue replacement by growing cells on the PNIPAAm-based culture supports [7]–[9]. Given that the thermally detached cell monolayers are usually fragile, the use of suitable delivery carriers is necessary for surgical manipulation of sheet grafts. To this end, the bioadhesive gelatin hydrogels have been designed by our group to transfer the corneal endothelial cell (CEC) sheets from the PNIPAAm coatings to the anterior chamber of the eye [10], [11]. However, it is worth noting that intracameral implantation of the gelatin carriers with dense structure may interrupt the transport of nutrients and upset the balance of intraocular pressure. Recently, we have introduced a simple stirring process combined with freeze-drying method for the development of gelatin hydrogels with enlarged pore structure that can improve the aqueous humor circulation [12]. These porous carriers exhibit better biocompatibility than those prepared by air-drying method.

Porous hydrogels are becoming more and more important as cell scaffold materials for tissue engineering applications [13]. During tissue formation/remodeling, the porous supporting structure of biomaterials can regulate nutrient uptake to and waste removal from the cultured cells. Additionally, the pore size and distribution are known to affect the stability of polymer matrices (i.e., mechanical strength or degradation resistance) [14]. Among the processes available for the fabrication of porous hydrogels, freeze-drying is one of the most effective methods to create numerous cavities within the bulk material. Investigators have shown that the pore size of scaffolds made of gelatin [15], collagen/hyaluronic acid [16], and poly(l-glutamic acid) [17] decreases with a decrease in the freezing temperature, probably due to the effect of heat transfer rate on the nucleation and growth of ice crystals. On the other hand, to enhance the performance of porous carriers, cross-linking is a technique to modify the material structure and texture. In our laboratory, the porous gelatin hydrogels were cross-linked with 1 mM 1-ethyl-3-(3-dimethyl aminopropyl) carbodiimide (EDC) for different time periods [18]. The earlier observations suggest that 12 h is the optimal cross-linking reaction time for preparation of cell sheet delivery carriers in the present case.

Porous gelatin scaffolds have seen many applications in tissue engineering and regenerative medicine [19]–[21]. In addition to both the freezing temperature and cross-linking time, the biopolymer concentration is a key to control the structure and function of porous hydrogels. To the best of our knowledge, the influence of solid content on the characteristics of cell sheet-based delivery carriers fabricated by a simple stirring process combined with freeze-drying method has not yet been found in the literature. Given that the safety of hydrogel carriers is critical to the success cell-mediated tissue regeneration, it is necessary to examine the biological responses to cross-linked porous gelatin materials before their use for corneal cell sheet engineering. In the present work, we aimed to investigate the effects of gelatin concentrations on the characteristics of cross-linked porous delivery carriers and their interaction with corneal endothelium. The aqueous gelatin solutions of different concentrations (5–15 wt%) were cooled to 25°C and stirred with a rate of 350 rpm for 20 min. To obtain the porous gelatin discs, the resulting solutions were frozen at −20°C for 24 h and lyophilized for 2 days. After cross-linking with 1 mM EDC for 12 h, the gelatin samples were characterized by scanning electron microscopy, porosity measurements, and ninhydrin assays. The mechanical and swelling tests, differential scanning calorimetry measurements, and glucose permeation studies were carried out to determine the relationship between biopolymer concentration and characteristics of cross-linked porous gelatin hydrogels. Cell-material interaction was also analyzed by monitoring the proliferation and gene expression of cultivated rabbit CECs exposed to various delivery carriers. Additionally, we performed an in vivo study in a rabbit model to examine the corneal endothelial tissue responses to these cross-linked porous carrier materials.

## Methods

### Ethics Statement

The animal use protocols (CGU10-043) were reviewed and approved by the Institutional Animal Care and Use Committee of Chang Gung University.

### Materials

Gelatin (Type A; 300 Bloom), 1-ethyl-3-(3-dimethyl aminopropyl) carbodiimide hydrochloride (EDC), ninhydrin reagent, glucose, and glucose assay kit (glucose oxidase/peroxidase reagent and o-dianisidine reagent) were purchased from Sigma-Aldrich (St. Louis, MO, USA). Balanced salt solution (BSS, pH 7.4) was obtained from Alcon Laboratories (Fort Worth, TX, USA). Phosphate-buffered saline (PBS, pH 7.4) was purchased from Biochrom AG (Berlin, Germany). Medium 199, gentamicin, Hanks’ balanced salt solution (HBSS, pH 7.4), trypsin-ethylenediaminetetraacetic acid (EDTA), and TRIzol reagent were purchased from Gibco-BRL (Grand Island, NY, USA). Collagenase type II was purchased from Worthington Biochemical (Lakewood, NJ, USA). Fetal bovine serum (FBS) and the antibiotic/antimycotic (A/A) solution (10,000 U/ml penicillin, 10 mg/ml streptomycin, and 25 µg/ml amphotericin B) were obtained from Biological Industries (Kibbutz Beit Haemek, Israel). All the other chemicals were of reagent grade and used as received without further purification.

### Preparation of Cross-linked Porous Gelatin Carriers

The aqueous gelatin solutions of different concentrations (5–15 wt%) were prepared by dissolution of gelatin powder in double-distilled water at 40°C. The 5 ml of resulting solutions were cooled to 25°C and stirred with a rate of 350 rpm for 20 min. After casting into the mold, the solutions were frozen at −20°C for 24 h and lyophilized at −55°C for 2 days. Then, the porous gelatin hydrogel sheets were further cross-linked with EDC by directly immersing the samples in an ethanol/water mixture (8∶2, v/v, pH 4.75) of 1 mM EDC. The cross-linking reaction was allowed to proceed at 25°C for 12 h, and the hydrogel sheets were thoroughly washed with double-distilled water to remove excess EDC and urea by-product. Subsequently, the cross-linked gelatin samples were frozen at −20°C for 24 h and lyophilized again. Using a 7-mm diameter corneal trephine device, the hydrogel sheets were cut out to obtain gelatin discs (∼700 µm in thickness). In this study, the disc samples prepared from 5, 10, and 15 wt% gelatin solutions were designated as G5, G10, and G15, respectively.

### Characterization of Cross-linked Porous Gelatin Carriers

Specimens were prepared for scanning electron microscopy (SEM) as described previously [22]. Small pieces of the hydrogel discs were cut off and mounted onto stubs using double-sided adhesive tape, and then gold coated in a sputter coater (Hitachi, Tokyo, Japan). The cross-section morphologies of the gelatin discs were examined using a Hitachi S-3000N SEM with an accelerating voltage of 10 kV. Twenty different pores were randomly selected, and the average pore diameters were calculated. Results were averaged on four independent runs.

The solvent replacement method was used for porosity measurements [12]. Each gelatin disc was first dried to constant weight (*W*_i_) in a vacuum oven. The test samples were immersed in absolute ethanol overnight, blotted with tissue paper to remove excess ethanol on the surface, and weighed (*W*_f_) immediately. The porosity (%) was calculated as ((*W*_f_-*W*_i_)/*Vρ*) × 100, where *V* is the volume of the hydrogel disc and *ρ* is the density of absolute ethanol. Results were averaged on four independent runs.

The ninhydrin assay was used to determine the amount of free amino groups of each porous gelatin disc. The test sample was weighed and heated with a ninhydrin solution for 20 min. After the test solution was cooled to room temperature and diluted in 95% ethanol, the optical absorbance of the solution was recorded with a UV-visible spectrophotometer (Thermo Scientific, Waltham, MA, USA) at 570 nm using glycine at various known concentrations as standard [23]. The amount of free amino groups in the gelatin disc before (*C*_i_) and after (*C*_f_) cross-linking is proportional to the optical absorbance of the solution. The degree of cross-linking of the gelatin discs was calculated as equation 1. Results were the average of five independent measurements.
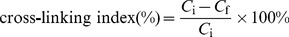
(1)


### Mechanical Tests

The gelatin sheets were placed in an environment with humidity of 75% for 24 h [24]. Then, the dumbbell-shaped samples were prepared by cutting wet sheets under pressure with a suitable mold. The gauge length of the specimens was 10 mm and the width was 5 mm. In order to determine the thicknesses without squeezing the test samples, another set of gelatin sheets with the same conditions were simultaneously prepared and immersed in liquid nitrogen for 10 min to keep the contents frozen. The thicknesses of these specimens could be obtained by measuring at three different points with a Pocket Leptoskop electronic thickness gauge (Karl Deutsch, Germany) and the average was taken for tensile testing. Young’s modulus of gelatin samples was determined from stress-strain curves using an Instron Mini 44 universal testing machine (Canton, MA, USA). All measurements were performed at 25°C and a relative humidity of 50% using a crosshead speed of 0.5 mm/min. Results were averaged on ten independent runs.

### Swelling Tests

The swelling ratio of each cross-linked gelatin sample was determined by immersing the hydrogel disc in BSS at 34°C (physiological temperature of the cornea) with reciprocal shaking (80 rpm) in a thermostatically controlled water bath. At specific time intervals, the swollen hydrogel discs were removed from the swelling medium, blotted with tissue paper to remove excess water on the surface, and weighed immediately. The swelling ratio of the test sample was calculated as equation 2, where *W*_s_ is the weight of the swollen gelatin hydrogel and *W*_i_ is its initial dry weight. Results were averaged on five independent runs.
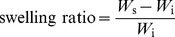
(2)


### Determination of Freezable Water Content

Differential scanning calorimetry (DSC) measurements were used to examine the states of water in the cross-linked gelatin discs. The samples were placed in a DSC cell (TA Instruments, New Castle, DE, USA), cooled to −20°C to freeze the swollen hydrogels, and heated to 20°C at a heating rate of 5°C/min under a nitrogen gas flow. The amount of freezable water was evaluated from the DSC endothermic ice-melting profile of the frozen hydrogel. The enthalpy of melting (Δ*H*_m_) obtained by integration and normalization is in unit of J/g of swollen hydrogel. Temperatures and enthalpies of melting of the samples were calibrated using pure water as the standard. The latent heat of water is 333.5 J/g of pure water. The gram of freezable water per gram of swollen gelatin hydrogel (*W*_fH_/*W*_s_) was calculated as Δ*H*_m_/333.5. Results were the average of six independent measurements.

### Glucose Permeation Studies

Glucose permeation studies were performed at 34°C using a horizontal glass diffusion cell (PermeGear, Hellertown, PA, USA) having two stirred chambers with sampling ports [25]. The donor chamber was filled with a 6.9 µmol/ml (the glucose concentration of aqueous humor in rabbit) glucose solution in BSS (3 ml) and receptor chamber with BSS (3 ml). After immersion in BSS until fully swollen, the cross-linked gelatin hydrogel samples were placed between the two chambers. During the measurements, all solutions were stirred continuously to provide uniform solute distribution and to reduce boundary layering of glucose. After 12 h, the receptor chamber was sampled and analyzed using a glucose assay kit, following the manufacturer’s instructions. Photometric readings at 540 nm were measured with a spectrophotometer (Thermo Scientific) and compared with a standard curve of known glucose concentrations. Results were averaged on six independent runs.

### Rabbit Corneal Endothelial Cell Cultures

All animal procedures were performed in accordance with the ARVO Statement for the Use of Animals in Ophthalmic and Vision Research. Twenty adult New Zealand white rabbits (National Laboratory Animal Breeding and Research Center, Taipei, Taiwan, ROC) were used for cell-material interaction studies. Primary rabbit CECs were prepared according to previously published methods [26]. Briefly, under a dissecting microscope (Leica, Wetzlar, Germany), Descemet’s membrane with the attached endothelium was aseptically stripped from the stroma and washed three times with PBS. The Descemet’s membrane-corneal endothelium complex was digested using 2 mg/ml collagenase in HBSS for 1 h at 37°C. Thereafter, the CECs were collected and resuspended in regular culture medium containing Medium 199 as a basal medium, 10% FBS, 50 µg/ml gentamicin and 1% A/A solution. Cultures were incubated in a humidified atmosphere of 5% CO_2_ at 37°C. Medium was changed every other day. Confluent monolayers were subcultured by treating with trypsin-EDTA for 2 min and seeded at a 1∶4 split ratio. Only second-passage cells were used for this study.

### Cell Viability and Proliferation Assays

Rabbit CECs (7×10^4^ cells/well) were seeded in 24-well plates containing regular growth medium and incubated overnight to allow cell attachment. After 1 week of cultivation, the sterilized gelatin discs were placed on the apical cell surface in direct contact with the confluent cultures. Rabbit CEC cultures without contacting disc samples served as control groups. Following incubation for 12 h, the cell viability was determined using the Live/Dead Viability/Cytotoxicity Kit from Molecular Probes (Eugene, OR, USA) [7]. This assay uses intracellular esterase activity to identify the living cells; the process cleaves the calcein acetoxymethyl to produce a green fluorescence. Ethidium homodimer-1 can easily pass through the damaged cell membranes of dead cells to bind to the nucleic acids, yielding a red fluorescence. After washing three times with PBS, the cultures were stained with a working solution consisting of 2 µL of ethidium homodimer-1, 1 mL of PBS, and 0.5 µL of calcein acetoxymethyl. Cells were then observed and imaged under fluorescence microscopy (Axiovert 200M; Carl Zeiss, Oberkochen, Germany). On the other hand, the cell growth was estimated using the CellTiter 96 Aqueous Non-Radioactive Cell Proliferation MTS Assay (Promega, Madison, WI, USA), in which MTS tetrazolium compound is bio-reduced by cells to form a water-soluble colored formazan [27]. A total of 100 µl of the combined MTS/PMS (20∶1) reagent was added to each well of the 24-well plate, and incubated for 3 h at 37°C in a CO_2_ incubator. The data of absorbance readings at 490 nm were measured using the Multiskan Spectrum Microplate Spectrophotometer (ThermoLabsystems, Vantaa, Finland). All experiments were performed in five replicates, and the results were expressed as relative MTS activity when compared to control groups.

### Quantitative Real-time Reverse Transcription Polymerase Chain Reaction and Western Blot Analyses

As mentioned above, the rabbit CECs were grown to confluence on 24-well plates in regular growth medium. After 12 h of direct contact between the cultures and sterilized gelatin discs, the total RNA was isolated from cells with TRIzol reagent according to the manufacturer’s procedure [28]. Reverse transcription of the extracted RNA (1 µg) was performed using ImProm-II (Promega) and Oligo(dT)_15_ primers (Promega). The sequences of the primer pairs for each gene are listed in Table 1. Quantitative real-time reverse transcription polymerase chain reaction (RT-PCR) was performed on a Light-Cycler instrument (Roche Diagnostics, Indianapolis, IN, USA) according to the manufacturer’s instructions with FastStart DNA Master SYBR Green I reagent (Roche Diagnostics). Each sample was determined in triplicate, and the gene expression results were normalized to the expression of glyceraldehyde-3-phosphate dehydrogenase (GAPDH).

**Table 1:** Sequences of primers used in gene expression analyses.

Genes^a^	Forward (5′–3′)	Reverse (5′–3′)
ATP1A1	GTCTTCCAGCAGGGCATGAA	TAAGGGCAACACCCATTCCA
VDAC2	CCACTGCTTCCATTTCTGCAA	CAGAGCAGACAGCGTGAGCTT
AQP1	GTGCTCACCCACAACTTCAACA	CATCGCCGTCCAGGTCATACT
GAPDH	TTGCCCTCAATGACCACTTTG	TTACTCCTTGGAGGCCATGTG

a ATP1A1: Na^+^,K^+^-ATPase alpha 1 subunit; VDAC2: voltage-dependent anion channel 2; AQP1: aquaporin 1; GAPDH: glyceraldehyde-3-phosphate dehydrogenase.

For the preparation of protein extracts, cells from each group were lysed in 1% NP-40 lysis buffer containing 1 mM EDTA, 1 mM ethylene glycol tetraacetic acid, 5 µg/ml antipain, 5 µg/ml pepstatin A, 1 mM phenylmethylsulfonyl fluoride, and 5 µg/ml aprotinin [11]. Protein concentrations were determined by protein assay (Bio-Rad, Hercules, CA, USA) and 50 µg of protein per lane was separated by electrophoresis under reducing conditions in 10% polyacrylamide gel with sodium dodecyl sulfate (SDS-PAGE). For Western blotting, SDS-PAGE gels were transferred to poly(vinylidene difluoride) membranes that were blocked with 5% nonfat milk in tris-HCl-buffered saline containing 0.1% Tween-20 (TTBS) for 1 h at room temperature. The membranes were then incubated with mouse anti-Na^+^,K^+^-ATPase alpha 1 subunit (1∶1000; Upstate Biotechnology, Lake Placid, NY, USA) primary antibodies with 5% nonfat milk in TTBS overnight at 4°C with gentle rocking. Next, blots were washed for three times with 0.1% TTBS solution and incubated with secondary antibodies conjugated to horseradish peroxidase (1∶5000; Chemicon International, Temecula, CA, USA) with 5% nonfat milk in TTBS for 1 h at room temperature. The SuperSignal West Pico chemiluminescent substrate (Pierce, Rockford, IL, USA) was used for detecting a secondary antibody on imaging films (Biomax MS, Eastman Kodak, Rochester, NY, USA). Anti-alpha-tubulin (1∶2000; Abcam, Cambridge, MA, USA) was used as loading controls.

### In vivo Studies

All animal procedures were performed in accordance with the ARVO (Association for Research in Vision and Ophthalmology) Statement for the Use of Animals in Ophthalmic and Vision Research. Twenty-four adult New Zealand white rabbits (National Laboratory Animal Breeding and Research Center), weighing from 3.0 to 3.5 kg and 16–20 weeks of age, were used for this study. Animals were healthy and free of clinically observable ocular surface disease. Surgical operation was performed in the single eye of animals, with the normal fellow eye. In the three test groups (G5, G10, and G15) of animals (six rabbits/group), the sterilized gelatin discs were inserted in the anterior chamber of the eye. The remaining six rabbits received no implant (only corneal/limbal incision) and served as a control group.

The rabbits were anesthetized intramuscularly with 2.5 mg/kg body weight of tiletamine hydrochloride/zolazepam hydrochloride mixture (Zoletil; Virbac, Carros, France) and 1 mg/kg body weight of xylazine hydrochloride (Rompun; Bayer, Leverkusen, Germany), and topically with two drops of 0.5% proparacaine hydrochloride ophthalmic solution (Alcaine; Alcon-Couvreur, Puurs, Belgium). After disinfection and sterile draping of the operation site, the pupil was dilated with one drop of 1% atropine sulfate ophthalmic solution (Oasis, Taipei, Taiwan, ROC), and a lid speculum was placed. Under the surgical microscope (Carl Zeiss, Oberkochen, Germany), the cornea was penetrated near the limbus by using a slit knife. Then, the corneal/limbal incision was enlarged to 7.5 mm with corneal scissors to allow the insertion of a cross-linked porous gelatin disc in the anterior chamber (See the Video S1). The incision site was finally closed with 10–0 nylon sutures.

To determine the implant-tissue interaction in the anterior chamber, the rabbits were anesthetized under the same conditions as for surgery. Ophthalmic evaluations were performed before and 3 days after surgical insertion of material implants. The CEC morphology and density in rabbit eyes was measured by specular microscopy (Topcon Optical, Tokyo, Japan). Each data point is an average of three independent observations.

### Statistical Analysis

Results were expressed as mean ± standard deviation. Comparative studies of means were performed using one-way analysis of variance (ANOVA). Significance was accepted with *P*<0.05.

## Results and Discussion

### Characterization of Cross-linked Porous Gelatin Carriers

**Figure 1:**
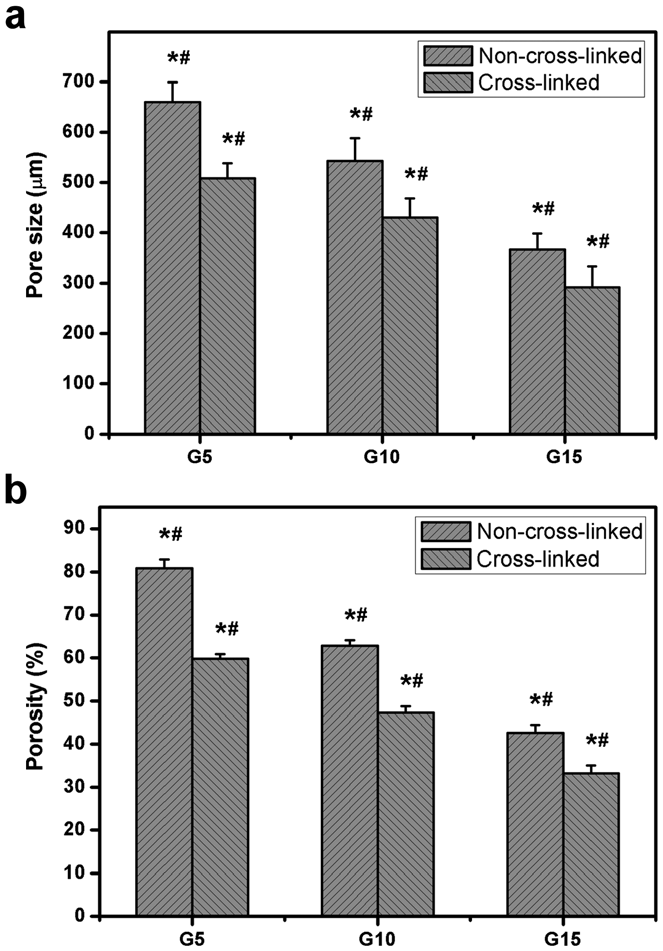
Characterization of porous structure. (a) Pore size and (b) porosity of various gelatin discs. An asterisk indicates statistically significant differences (*P<0.05; *n* = 4) between the non-cross-linked and cross-linked groups for each type of gelatin disc. ^#^ *P*<0.05 versus all groups (compared only within non-cross-linked or cross-linked groups).

The pore size and porosity of various gelatin hydrogel discs are shown in Fig. 1. The non-cross-linked samples from G5, G10, and G15 groups exhibited the pore size of 660±39, 543±45, and 367±31 µm, respectively. The values showed significant differences between these three groups (*P*<0.05). In addition, the porosity was significantly higher in the G5 groups (80.9±2.0%), compared with those of the G10 (62.8±1.3%) and G15 groups (42.6±1.8%; *P*<0.05). These data demonstrate that the biopolymer concentration has potential to influence the porous structure of hydrogel carriers. Wu et al. have reported that at a gelatin concentration of 1% (g/ml), the scaffolds fabricated by unidirectional freeze-drying method had a microtubule-like orientation porous structure with a width ranging from 100 to 200 µm, and a length from 200 to 500 µm [29]. However, both the pore width and length decreased to ∼40 µm when the gelatin concentration was increased to 5%, probably due to the effect of gelatin concentration on the growth of ice crystals during the freeze stage. In accordance with this earlier study, we also observed decrease in pore size of gelatin carriers with increasing solid content. In 2007, Van Vlierberghe et al. have synthesized porous hydrogels by chemical modification of gelatin with methacrylamide side groups and UV-induced photopolymerization as well as cryogenic treatment [30]. The porosity significantly decreased from 96 to 78% with increasing gelatin concentration in the range of 5–15% (w/v). Although at the same gelatin concentration, the solutions used for preparing the hydrogel discs were handled by a different procedure (stirring and freeze-drying). Hence, in the present work, the measured porosity results were not comparable to the literature.

**Figure 2:**
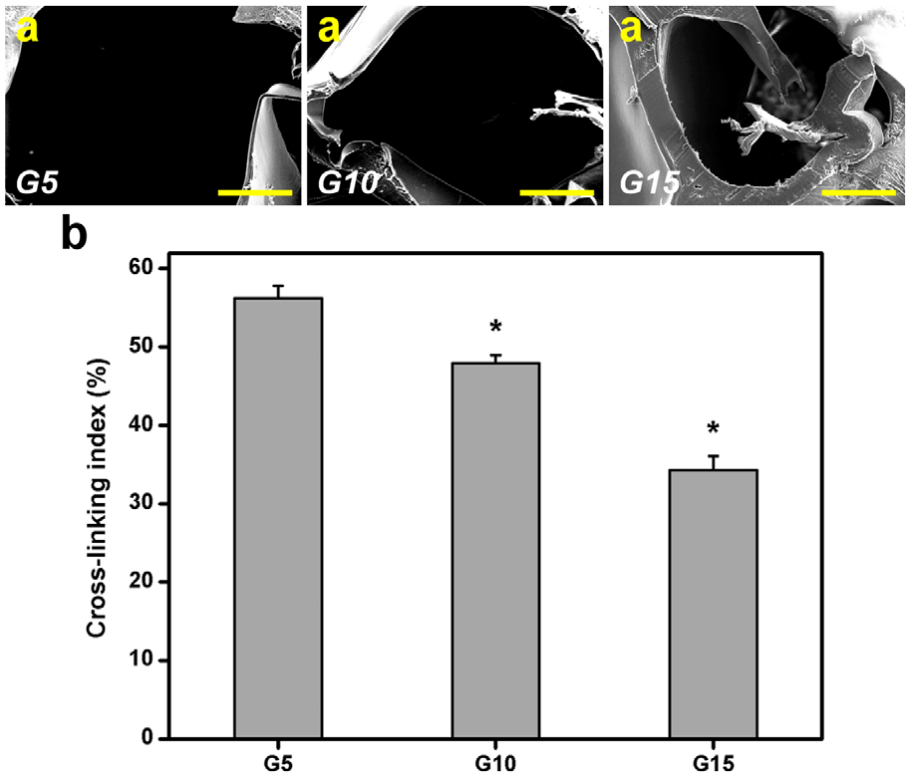
Morphological observations and cross-linking analyses. (a) Representative scanning electron microscopic images of various gelatin discs after cross-linking. Scale bars: 100 µm. (b) Cross-linking index of various gelatin discs. An asterisk indicates statistically significant differences (*P<0.05; *n* = 5) as compared with the G5 groups.

On the other hand, a similar trend was noted for the effect of biopolymer concentration on porous structure variation of EDC cross-linked hydrogels (Fig. 1). As shown in Fig. 2a, each test sample maintained its porous architecture after cross-linking. However, the chemical modification of gelatin discs resulted in different levels of changes in porous characteristics. For the G5 groups, the pore size and porosity of carbodiimide cross-linked hydrogels were respectively reduced by about 152 µm and 21.1% as compared to those before cross-linking. In contrast, for the G15 groups, significantly less decrease in the aforementioned two parameters was observed before and after treatment of gelatin with cross-linkers. These results indicate that the biopolymer concentration may play an important role in regulating the cross-linking reaction. Fig. 2b shows the cross-linking index of various test samples. When the gelatin concentration was increased from 5 to 15 wt%, the extent of cross-linking of porous discs significantly decreased (*P*<0.05). Our previous report has demonstrated that as compared to the discs with dense structure, the porous carriers more effectively increase the contact area between the gelatin and EDC, thereby leading to a higher cross-linking degree [12]. The findings of this study suggest that the large pore size and high porosity of the hydrogel materials enhance the collision frequency of biomacromolecules with chemical cross-linkers, which possibly promotes the formation of cross-links between lysine and glutamic acid residues on gelatin chains.

### Mechanical Tests

**Figure 3:**
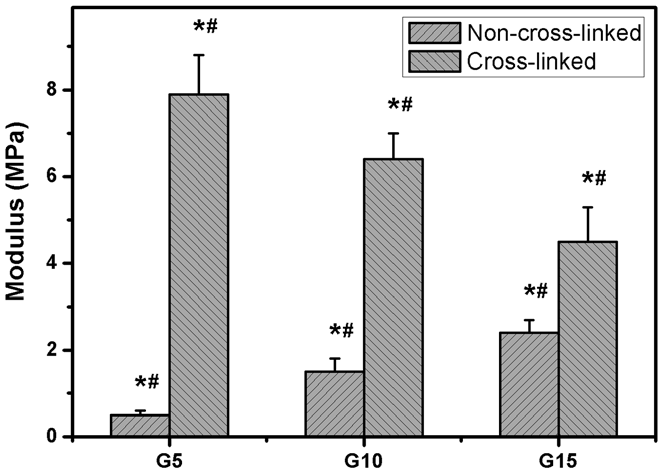
Mechanical tests. Young’s modulus of various gelatin samples. An asterisk indicates statistically significant differences (*P<0.05; *n* = 10) between the non-cross-linked and cross-linked groups for each type of gelatin sample. ^#^ *P*<0.05 versus all groups (compared only within non-cross-linked or cross-linked groups).

Given that the gelatins are hygroscopic, the wet membranes have been shown to possess significantly lower ultimate tensile strength and higher stretchability than their dry counterparts [24], [31]. In order to examine the stability of hydrogel materials, the mechanical properties of porous gelatin sheets were investigated by tensile tests. Fig. 3 summarizes the Young’s modulus of various gelatin samples. For the non-cross-linked gelatin groups, the Young’s modulus significantly increased from 0.5 to 2.4 MPa with increasing gelatin concentration in the range of 5–15% (w/v) (*P*<0.05), suggesting the influence of biopolymer concentration. Chung et al. have prepared highly porous, three-dimensional sponge composed of Ca-alginate and galactosylated chitosan and indicated that the mechanical property of the sponge is enhanced with an increase of the content of galactosylated chitosan [32]. A study from Goh et al. also observed the effect of the initial polymer concentration on the mechanical behavior of the poly(l-lactide) foams [33]. While the 1 wt.% solution gave rise to soft porous scaffolds with high flexibility, the higher concentration solutions (3–5 wt.%) gave rigid foams. Our present results are compatible with their findings. However, after cross-linking treatment, it was shown that the inverse effect occurred. The measured modulus of gelatin sample G10 was significantly lower than the Young’s modulus for sample G5 (*P*<0.05), but significantly higher than for sample G15 (*P*<0.05). These data indicate that the mechanical properties of porous gelatin carriers are also greatly dependent on the degree of cross-linking, controlled by the solid content (as mentioned above). The gelatin molecules contained a larger amount of cross-links may increase the material stability of delivery carriers.

### Swelling Tests

**Figure 4:**
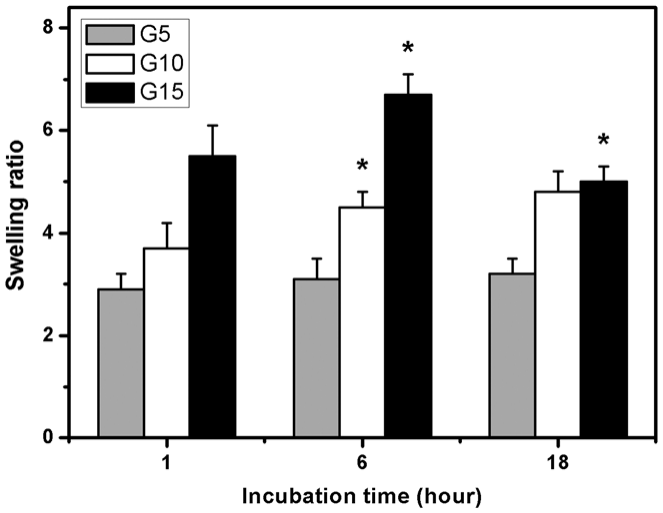
Swelling tests. Time course of swelling ratio of various gelatin discs after incubation in BSS at 34°C. An asterisk indicates statistically significant differences (*P<0.05; *n* = 5) for the mean value of swelling ratio compared with the value at the previous time point.

Hydrogel swelling is one of the most attractive features for the production of carrier materials in the intraocular delivery of bioengineered CEC sheets [34]. The gelatin hydrogels should swell rapidly to a size sufficient to facilitate the attachment of cell grafts onto corneal posterior surfaces. In the present work, the water absorption capability was evaluated by monitoring the swelling ratio of various gelatin discs as a function of incubation time. As shown in Fig. 4, the test samples from G5 groups exhibited the swelling ratio ranging from 2.9 to 3.2 within 18 h, indicating no significant difference in water uptake at three different times (*P*>0.05). In the G10 groups, the swelling ratio reached a plateau level of 4.5±0.3 within 6 h of incubation in BSS at 34°C. It was noted that although the gelatin sample G15 swelled rapidly at 1 h, the hydrogel disintegrated into small fragments (Fig. S1). Our findings suggest that the cross-linked porous gelatin discs prepared from high biopolymer concentration (i.e., 15 wt%) seem unsuitable for use as CEC carriers due to their rapid dissolution in aqueous environments and potential squeezing on the anterior segment tissues. On the other hand, the order of increasing swelling ratio for the discs at each time point from 1 to 6 h was G15> G10> G5. One possible explanation for these observations is that the formation of higher number of covalent cross-links between adjacent polymer chains may cause an elastic network retraction force against additional swelling of gelatin hydrogels [12].

### Determination of Freezable Water Content

**Figure 5:**
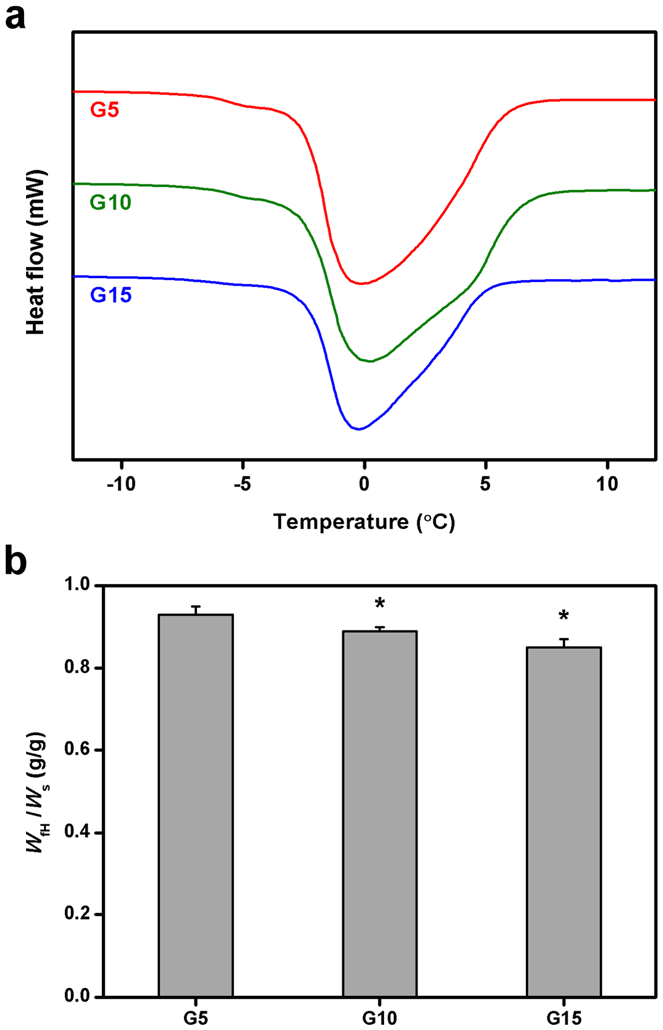
Determination of freezable water content. (a) Typical DSC thermograms of swollen gelatin hydrogel discs. (b) Freezable water content (*W*_fH_/*W*_s_) of various gelatin samples. An asterisk indicates statistically significant differences (*P<0.05; *n* = 6) as compared with the G5 groups.

Given the importance of solute permeability of hydrogels, it is highly desired to determine the content of freezable water that represents the fraction of water available for solute diffusion. Fig. 5a illustrates the representative DSC thermograms of swollen gelatin hydrogels. Only one melting peak was noted in the curves for G5, G10, and G15 groups at around 0°C. The findings are consistent with our previous observation that the major part of water in porous gelatin discs prepared by a simple stirring process combined with freeze-drying method is in the form of free water [12]. These water molecules in the center of pores and the space between network chains are almost freely mobile, which may minimize resistance to solute diffusion. Fig. 5b shows the total amount of freezable bound water and free water in various swollen gelatin hydrogels. The freezable water content in the G5, G10, and G15 groups was 0.93±0.02, 0.89±0.01, and 0.85±0.02, respectively. The values showed significant differences between these three groups (*P*<0.05). Our findings suggest that the mobile fraction of water in the hydrogel discs is highly correlated with the gelatin concentration. It has been reported that due to either porosity confinement or interaction, the freezable water is depressed in a polymer-water matrix [35]. To overcome the drawbacks associated with low freezable water content caused by the dense structure of air-dried disc samples, we have fabricated the highly porous gelatin materials by using a simple stirring process and freeze-drying method. The study reported here was designed to further measure the impact of biopolymer concentration on the freezable water content of cross-linked porous gelatin matrices. Since the water molecules in swollen hydrogel discs may be interacted with free carboxylic acid and amino groups of gelatin by hydrogen bonding, the existence of a significantly larger amount of nonfreezable bound water is noted for the samples with a higher solid content. In particular, for the discs in the G15 groups, the number of remaining free carboxylic acid and amino groups significantly increased attributed to the low cross-linking efficiency.

### Glucose Permeation Studies

**Figure 6:**
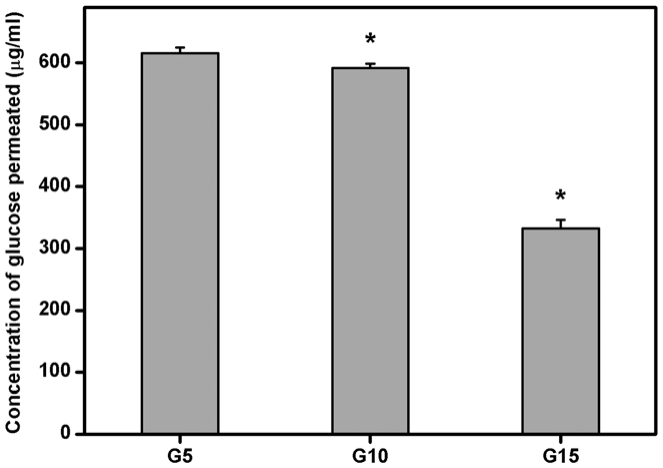
Glucose permeation studies. Concentration of glucose permeated through various gelatin discs at 34°C. An asterisk indicates statistically significant differences (*P<0.05; *n* = 6) as compared with the G5 groups.

The continued residence of biomaterial implants in the ocular anterior chamber may disturb aqueous humor dynamics and interrupt nutrient transport processes. Therefore, the nutrient permeability of carrier materials was investigated by using glucose (i.e., a major source of nutrients in the aqueous humor) as a probe molecule [18]. The concentration of glucose permeated through various gelatin hydrogel discs at 34°C is shown in Fig. 6. In the G5 groups, the detected glucose concentration was 615.5±9.2 µg/ml, which was significantly higher than those in the G10 (591.3±7.4 µg/ml) and G15 (332.5±13.6 µg/ml) groups (*P*<0.05). The present data indicate that the amount of permeated nutrients in the gelatin samples can be reduced due to the decrease of pore size and porosity. It has been reported that the pore wall thickness of poly(vinyl alcohol) scaffolds can be controlled by varying the polymer solution concentration [36]. Mao et al. have demonstrated that the thickness of pore wall for the chitosan-gelatin hybrid polymer network scaffolds from 3.5 wt% solution is thicker than that from 0.85% [37]. As observed by the SEM, the sample G15 presented higher pore wall thickness than that of other gelatin materials (Fig. 2a). The thickness of obtained pore wall may be one of the reasons for the differences in glucose diffusion rates between samples with different gelatin contents. Additionally, the lowest fraction of mobile water may simultaneously limit solute permeation through the gelatin sample G15. When the biopolymer concentration is decreased to 10 wt%, the hydrogel discs have lower resistance to glucose transport.

### Cell Viability and Proliferation Assays

**Figure 7:**
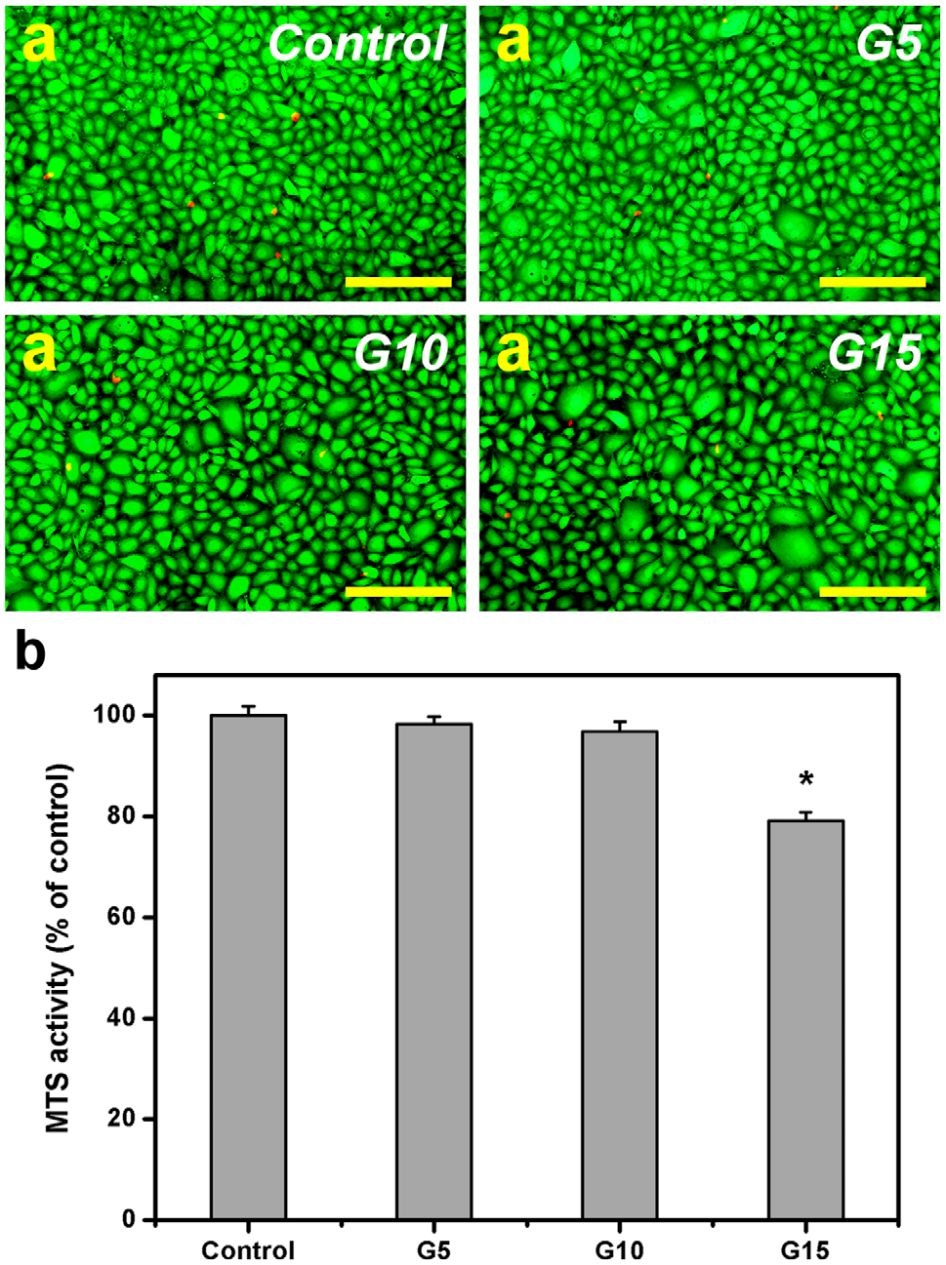
Cell viability and proliferation assays. (a) Cell viability of rabbit corneal endothelial cell cultures was determined by staining with Live/Dead Viability/Cytotoxicity Kit in which the live cells fluoresce green and dead cells fluoresce red. Fluorescence images of cells in controls (without gelatin materials) after 12 h of direct contact with different types of gelatin samples. Scale bars: 100 µm. (b) Cell proliferation assay of rabbit corneal endothelial cell cultures after 12 h of direct contact with various gelatin samples. Results are expressed as percentage of control groups (MTS activity of cells cultured in the absence of gelatin materials). An asterisk indicates statistically significant differences (*P<0.05; *n* = 5) as compared with the control groups.

The information from cell-material interactions will facilitate the engineering of the appropriate gelatin hydrogels as cell sheet delivery carriers. In this study, the biocompatibility of the hydrogels was investigated by means of Live/Dead assays. Fig. 7a is a representative photograph of rabbit CECs labeled with Live/Dead stain, where the live cells fluoresce green and the dead cells fluoresce red. The majority of control cultures were viable. Prominent green fluorescence was observed for all the test groups, indicating a large percentage of live cells. In addition, only a few red-stained nuclei were found. The results suggest that these cross-linked porous gelatin discs do not cause significant cytotoxicity. Despite exhibiting good cytocompatibility, the hydrogels with different porous structures have been reported to have altered cell proliferation [12]. Therefore, quantitative analysis for rabbit CEC growth was performed following the cell proliferation MTS assays, and the results are shown in Fig. 7b. After 12 h of cultivation, similar levels of mitochondrial dehydrogenase activity (MTS activity) were observed in the control, G5, and G10 groups and not statistically different (*P*>0.05). By contrast, the MTS activity was significantly reduced by about 21% (*P*<0.05) for the gelatin sample G15 as compared to those of the control groups. Our data demonstrate that the rabbit CECs in the G15 groups are less metabolically active than those from the G5 and G10 groups. The noticeably distinct cell growth behavior is attributed to the influence of biopolymer concentration. However, the effects of other parameters such as mechanical strength, porosity, and nutrient permeation also seem to play a vital role in modulating cell proliferation. Peyton et al. have used poly(ethylene glycol) hydrogels to investigate the impact of extracellular matrix mechanics on smooth muscle cell behavior and concluded that the cells proliferate more quickly when exposed to stiffer microenvironments [38]. In addition, it has been reported that in the low porosity conditions for poly(α-hydroxyl acids)-based composites fabricated by phase separation, the porous polymer matrices are not ideal for cell survival and proliferation owing to mass transport limitations [39].

We have previously investigated the effects of charge [10], molecular weight [10], Bloom index [24], [40], and cross-linker type [41], [42] on the proliferation of different types of ocular cells (corneal endothelial, iris pigment epithelial, and retinal pigment epithelial cells). However, these reports adopt an indirect contact methodology to examine the cell-material interactions. Given that the direct contact between biomaterial carriers and cell sheets is essential for transplantation of bioengineered CEC monolayers, the present work explores cell growth in the presence of gelatin hydrogel discs without placing the culture inserts. The test will more accurately reflect the MTS activity levels in the cell sheet grafts at the time of manipulation with delivery carriers. On the other hand, the ionic interactions between the test materials and nutrients in culture media are known to affect the regulation of cell growth [25]. Since the gelatin is zwitterionic and carries both free carboxyl and amine functionalities, the strength of ionic interactions may depend on the functional groups in this biomaterial. A high solid content for the discs in the G15 groups leads to strong ionic interaction and insufficient nutrient availability, thereby inhibiting the proliferation of cultured CECs.

### Quantitative Real-time Reverse Transcription Polymerase Chain Reaction and Western Blot Analyses

**Figure 8:**
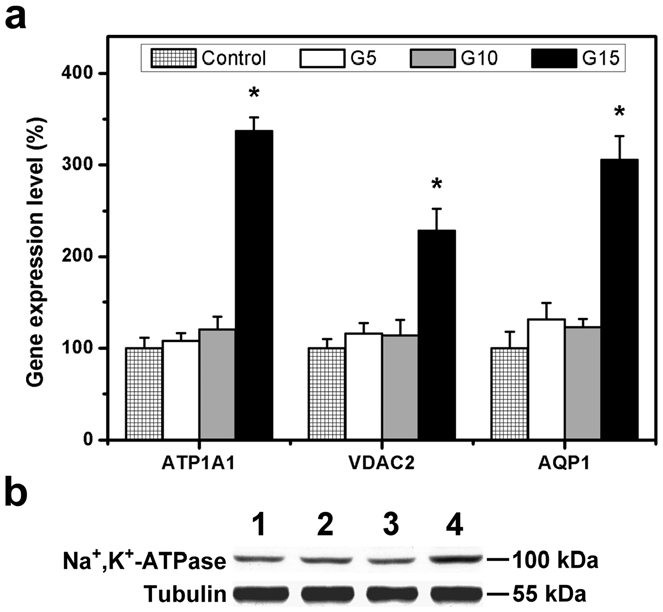
Quantitative real-time reverse transcription polymerase chain reaction and Western blot analyses. (a) Gene expression level of ATP1A1, VDAC2, and AQP1 in rabbit corneal endothelial cells after 12 h of direct contact with various gelatin samples, measured by real-time RT-PCR. Normalization was done by using GAPDH. Data in the experimental groups are percentages relative to that of control groups (cells cultured in the absence of gelatin materials). An asterisk indicates statistically significant differences (*P<0.05; *n* = 3) as compared with the control groups. (b) Western blot analysis of Na^+^,K^+^-ATPase expression in the rabbit corneal endothelial cells after 12 h of direct contact with gelatin samples. Lane 1: control (without gelatin materials), Lane 2: G5, Lane 3: G10, and Lane 4: G15 groups.

In our laboratory, the gelatin carriers with enlarged pore structure have been developed to minimize adverse effects on corneal physiology [12]. Here, we performed a study aiming at evaluating the function of rabbit CECs after 12 h of direct contact with various gelatin hydrogel discs. The gene expressions of membrane transport proteins including Na^+^,K^+^-ATPase alpha 1 subunit (ATP1A1), voltage-dependent anion channel 2 (VDAC2), and aquaporin 1 (AQP1) were analyzed by using a quantitative real-time RT-PCR (Fig. 8a). In the control groups, the detected expression level for each gene was defined as 100%. The CECs exposed to the gelatin samples G5 and G10 showed similar expression profiles for these three transcripts. However, the cultures of G15 groups had significantly higher ATP1A1, VDAC2, and AQP1 mRNA levels than did those of all the other groups (*P*<0.05). In particular, the ATP1A1 expression reached the highest value for the group of G15 with 311% amplification over the G5 counterparts. Western blotting was performed in order to determine whether the alterations in gene expressions were correlated to altered protein levels (Fig. 8b). Western blot analysis with a monoclonal antibody for Na^+^,K^+^-ATPase alpha 1 subunit demonstrated a 100-kDa band in rabbit CECs. The cultures from control, G5, and G10 groups had similar protein expression levels, suggesting the intact ionic pump functions. However, for the G15 groups, the protein bands were more intense in the lane. These results indicate that the ionic pump function of cultured CECs is abnormal following exposure to the cross-linked porous hydrogel discs prepared from high gelatin concentration.

In vivo, the corneal endothelium is a thin cell monolayer that forms the posterior boundary of the cornea [43]. The pump-leak mechanism is one of the most important functions in the endothelium responsible for maintaining corneal transparency and hydration [44]. It has been documented that the number of pump sites per cell increases with decreasing bovine CEC density [45]. In order to provide the required pumping capacity of the corneal endothelium at low cell density, the gene expressions of membrane transport proteins may be increased appropriately. As mentioned in the previous sections (*Glucose permeation studies* and *Cell viability and proliferation assays*), the gelatin hydrogels with a high solid content possibly reduce the access of nutrients to the surrounding cells and cause a decrease in the number of proliferated CECs. Hence, the high expression of ion channel and pump genes is observed in the cultures of G15 groups, indicating abnormal transmembrane transport. Although this phenomenon has not been considered in the literature, our findings suggest that the cross-linked porous carriers prepared from gelatin concentration of 5–10 wt% are not detrimental to the maintenance of normal ATP1A1, VDAC2, and AQP1 expressions.

### In vivo Studies

**Figure 9:**
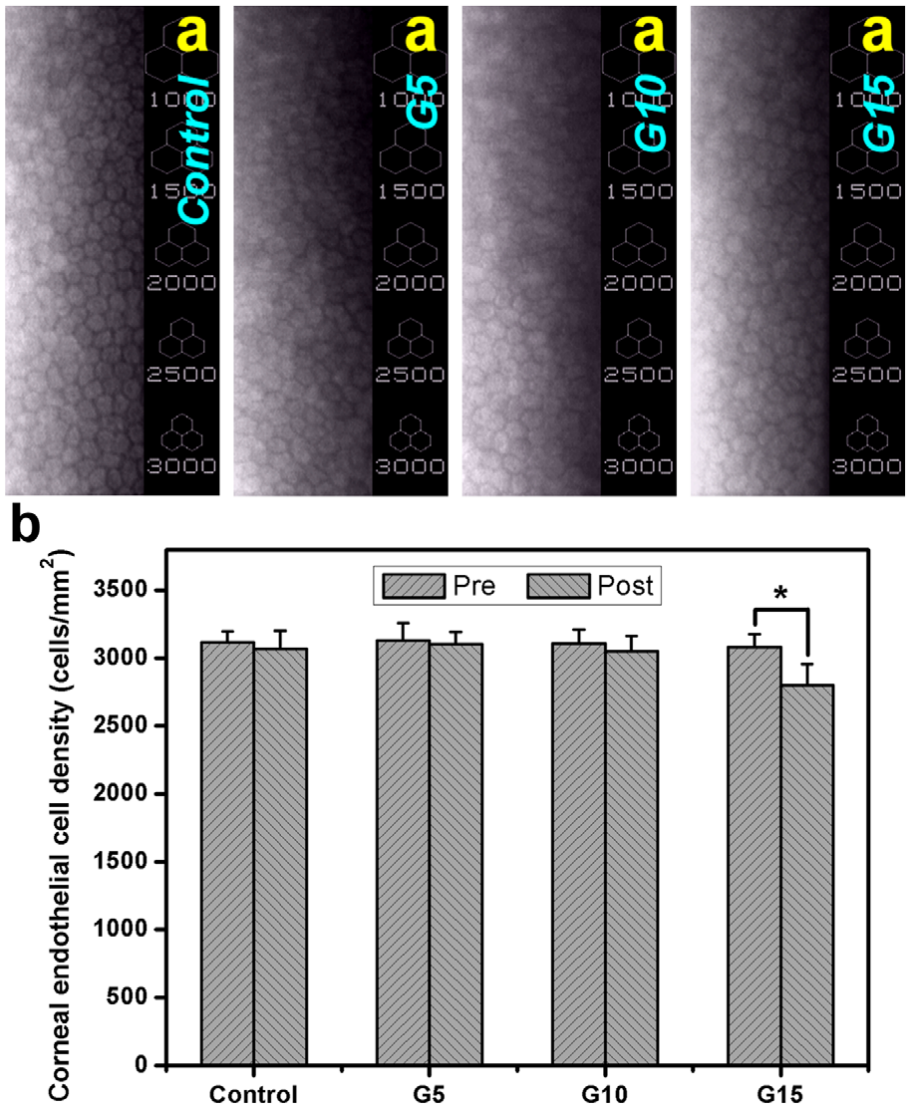
In vivo studies. Specular microscopy measurements of rabbit corneal endothelium 3 days after surgical insertion of various gelatin implants in the ocular anterior chamber. (a) Typical images; (b) graph of corneal endothelial cell count. An asterisk indicates statistically significant differences (*P<0.05; *n* = 6) between the preoperative (Pre) and postoperative (Post) cell density for each type of gelatin disc. The rabbits received no implant (only corneal/limbal incision) and served as a control (sham-operated) group.

For the corneal endothelium, the cellular hexagonality is a sensitive and reliable marker of tissue damage. Specular microscopy is a noninvasive test to assess endothelial cell abnormality and loss related to elevated intraocular pressure during glaucoma diagnostics [46]. Furthermore, the corneal endothelial monolayer surrounding the carbodiimide cross-linked gelatin implants exhibits significantly higher percent hexagonality than did those of rabbits bearing the glutaraldehyde treated hydrogels [42]. Here, the safety aspects of cell carrier materials for intraocular delivery use were investigated via the effects on CEC loss. Fig. 9a illustrates the representative specular microscopic images of rabbit corneal endothelium 3 days after surgical insertion of various gelatin hydrogel discs in the ocular anterior chamber. No change in cellular hexagonality was noted for the control and all the experimental groups. Our findings suggest that the sham operation (i.e., only corneal/limbal incision) and the intracameral implantation of cross-linked porous gelatin carriers do not affect the corneal endothelial morphological characteristics.

The results of quantitative specular microscopic analysis are shown in Fig. 9b. In the control groups, the mean postoperative rabbit CEC density was 3069±135 cells/mm^2^, which was similar to that found before surgery (*P*>0.05). No significant differences were also found in the endothelial cell count of G5 (3104±88 cells/mm^2^) and G10 (3053±109 cells/mm^2^) groups compared with their respective values at preoperation (*P*>0.05). However, in the G15 groups, the preoperative and postoperative cell density were 3080±95 and 2801±156 cells/mm^2^, respectively, indicating that the mean change is −9.1%. Although we were unable to detect a statistically significant change in postoperative CEC density between the control and G15 groups, a 3-day exposure to this type of gelatin hydrogel disc causes a reduction in cell count. In the current study, the effects of hydrogel carriers on the corneal endothelial morphology and count are evaluated in relation to biopolymer concentration. Our findings further corroborate the relationship between the CEC density and pump function in animals bearing various cross-linked porous gelatin materials.

### Conclusions

Although we have previously introduced a simple stirring process combined with freeze-drying method for the development of porous gelatin discs, the effect of biopolymer concentration on the characteristic and safety of hydrogel carriers has not been explored in that report. By controlling the amount of gelatin in aqueous solutions, the resulting disc samples exhibit varying porous structures and degrees of cross-linking, which greatly affects their Young’s modulus and swelling ratio. When the biopolymer concentration is in the range of 5–10 wt%, the hydrogels have high freezable water content (0.89–0.93) and concentration of permeated glucose (591.3–615.5 µg/ml). These features are beneficial to the in vitro cultivation of CECs without limiting proliferation and changing expression of ion channel and pump genes. In vivo studies by analyzing the rabbit CEC morphology and count also demonstrate that the implanted gelatin discs with the highest solid content may cause unfavorable tissue-material interactions. The cross-linked porous carriers prepared from suitable biopolymer concentrations can be further tested by the intraocular delivery bioengineered cell sheets in an animal model.

## Supporting Information

Figure S1 **Gross observations.** Typical photographs of gelatin sample G15 are shown (a) before testing and (b) after incubation in BSS at 34°C for 1 h.
(TIF)

Video S1 **Surgery.** Implantation of a cross-linked porous gelatin disc in the anterior chamber.
(WMV)
