# Supplementary figures and images for: Characterization of Cross-Linked Porous Gelatin Carriers and Their Interaction with Corneal Endothelium: Biopolymer Concentration Effect
Source: PLoS One. 2013 Jan 30;8(1):e54058. doi: 10.1371/journal.pone.0054058 (PMC3559727; doi:10.1371/journal.pone.0054058)

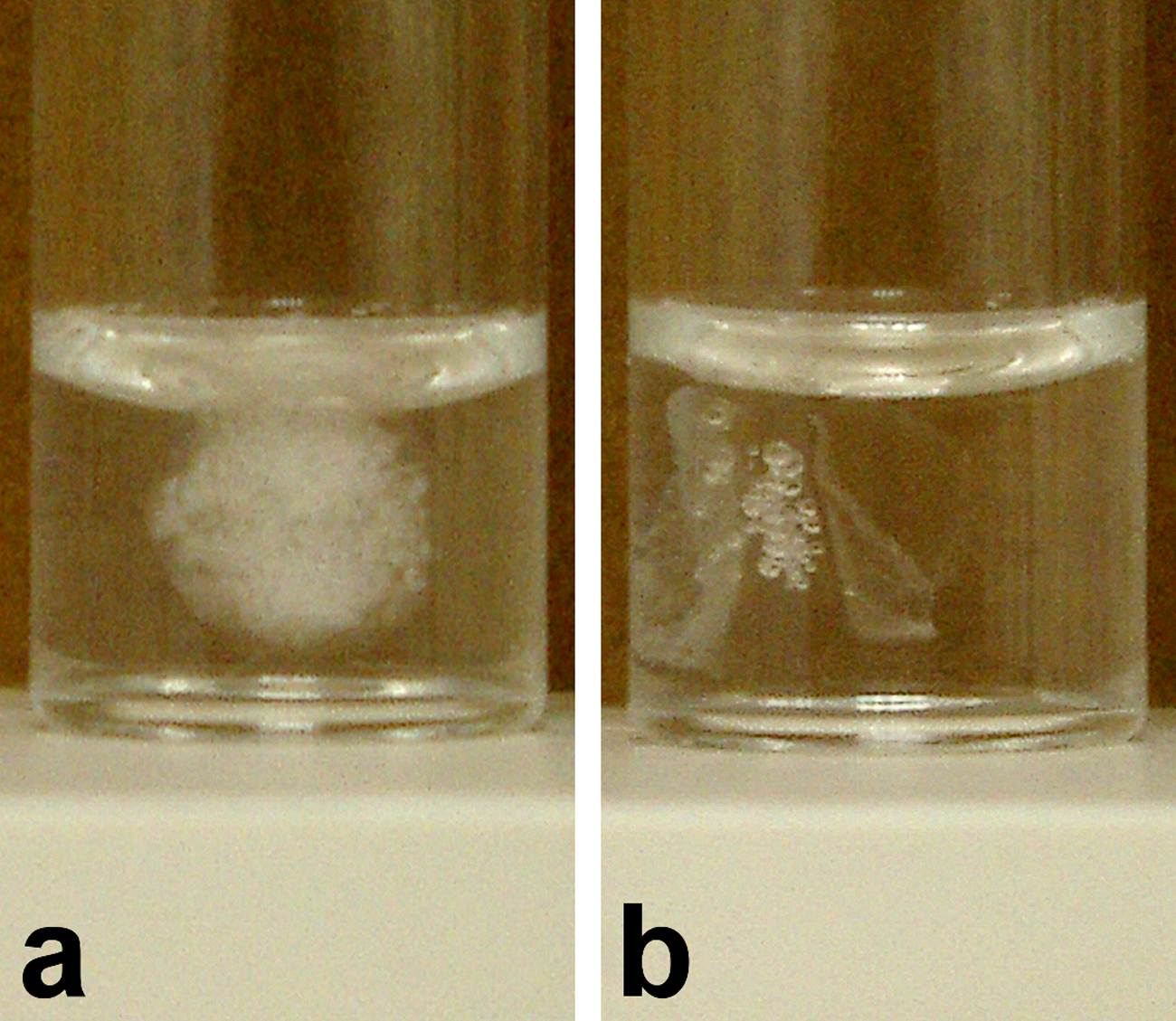

Supplement: Figure S1 — Gross observations. Typical photographs of gelatin sample G15 are shown (a) before testing and (b) after incubation in BSS at 34°C for 1 h. (TIF) [file pone.0054058.s001.tif]
